# Capacity Building Using Digital Technology for Occupational Therapists and Caregivers in Pakistan: A Participatory Action Research Approach

**DOI:** 10.5195/ijt.2022.6509

**Published:** 2022-12-13

**Authors:** Rukaiya Yawar, Zaheeruddin Asif

**Affiliations:** Institute of Business Administration, Karachi, Pakistan

**Keywords:** Caregiver, Developmental disabilities, Occupational therapy, Telehealth, Therapeutic communication

## Abstract

This paper describes the development and implementation of a telehealth system in Pakistan to build capacity of healthcare service providers and caregivers of children with developmental disabilities. An asynchronous telehealth system, in the form of a web app, improved therapy-related communication between the therapists and caregivers, thus enabling capacity building through sustained communication among the stakeholders. Participatory Action Research (PAR) identified barriers associated with communication, knowledge transfer, and caregiver learning. Data were collected via observations, interviews, focus groups, and field notes. The experiences of therapists and caregivers were analyzed to design and develop a system that works as a learning mechanism for caregivers in their native languages. The system also addresses socio-economic, geographic, and communication barriers as well as pandemic-imposed obstacles.

About 95% of global 52.9 million children less than 5 years of age, suffering from some form of a development disability, reside in Low and Middle Income Countries (LMICs) (Salomone et al., 2019), with almost 1.8 million of such children living in Pakistan (Olusanya et al., 2018). Children with a developmental disability also experience physical, behavioral and social problems broadly, which lead to learning and other challenges (Reichow et al., 2014). These children require therapies and support that are usually provided at rehabilitation centers, hospitals, and special schools (Bhat et al., 2021; Mishra & Siddharth, 2018).

Pakistan lags in establishing a structured medical and healthcare system. There is approximately only one rehabilitation professional for a population of one thousand people (Zahid et al., 2017). There are only a few rehabilitation units in large cities, and resources to build new centers in low-income locations are scarce (Hamdani et al., 2014). Thus, the care of children with developmental disability is primarily assumed by caregivers. Unfortunately, there are unique educational, economic, cultural, and geographical challenges in Pakistan that make access to care difficult for these children and their caregivers (Khan, 2020).

With the spread of the COVID-19 pandemic, telehealth options were rapidly adopted worldwide (Camden & Silva, 2021), and were introduced in a few care centers in Pakistan as well (Nadeem et al., 2020). Successful telehealth programs in high income countries are based on costly, high-speed internet solutions, which are not suitable for low and middle income countries (Borhani-Haghighi et al., 2020) including Pakistan.

Because there is an acute shortage of therapists and healthcare-providers in developing countries, the World Health Organization (WHO) recommends enlisting the help of parents as caregivers in the provision of therapy services at home (Salomone et al., 2019). However, this requires imparting therapy skills to the caregivers.

Appropriate telehealth services in conjunction with parental support can be a suitable avenue in Pakistan to provide health care to most of the rural population living far from the few specialized settings in urban areas. To explore this avenue, we posed the following research question:

*How can a low-cost asynchronous web-based application be developed and used to facilitate communication and caregiver learning, and support capacity building at different levels of a healthcare organization?*

Consequently, we developed “Sehat Agahi,” an easy to use, web-based communication app for therapists and caregivers of children affected by cerebral palsy. The aim is to support the trend toward developing a digital health care system and health care equity.

In this paper we present Sehat Agahi (meaning Health Awareness) and discuss its potential for supporting communications and capacity building in a healthcare organization and promoting healthcare equity.

This paper has three main sections. The next section on methodology discusses the context, participants, data collection, and analysis methods, as well as a detailed description of the web app, Sehat Agahi. The third section analyzes and discusses the app's impact on service redesign. The paper concludes by making recommendations and discussing the potential future role of the application in Pakistan's health sphere to facilitate the digitization of healthcare.

## Method and Materials

The development of Sehat Agahi was possible due to the confluence of three enabling factors. First, there was widespread availability of inexpensive smart phones across the country (Harris et al., 2020; Miller, 2020). Second, there was encouragement by WHO to enlist the help of caregivers by teaching them skills necessary to support their children in attaining physical milestones and developing positive behaviors (Salomone et al., 2019). Third, there was the realization that institutional capacity building can be enhanced by improving communication through the use of technology and external research collaboration (Khan, 2020; Miller, 2020).

**Figure 1:**
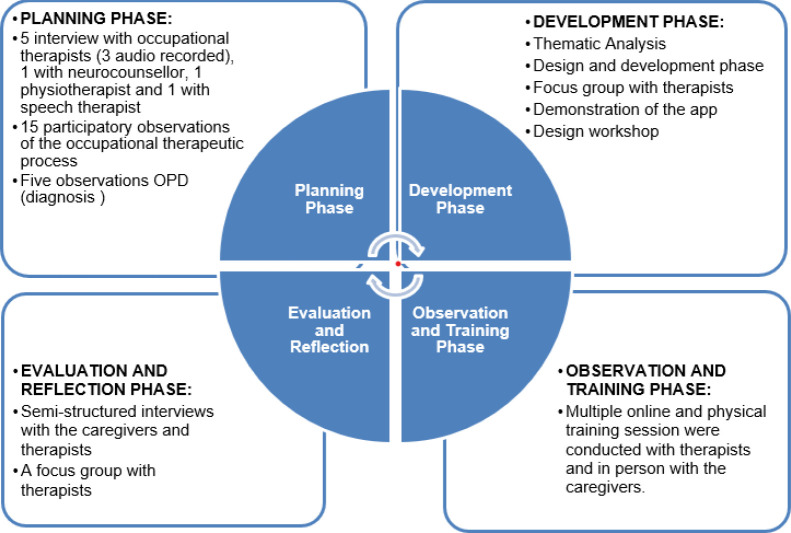
Four Phases of Participatory Action Research

Since, capacity building involves leveraging skills and knowledge from multiple disciplines (Kislov et al., 2014), we utilized Participatory Action Research (PAR). Participatory design is about engaging different stakeholders - in our case therapists, patients, caregivers, staff, and researchers - to develop a solution that satisfies the needs of all the stakeholders involved. We conducted this research in an iterative manner, involving several phases as depicted in Figure 1. Participating in the actual context and therapeutic process helped us to identify communication enhancement opportunities in everyday practice for the therapist. Participatory action research helped us to redefine services and fulfill the expectations based the stakeholders' needs.

**Figure 2:**
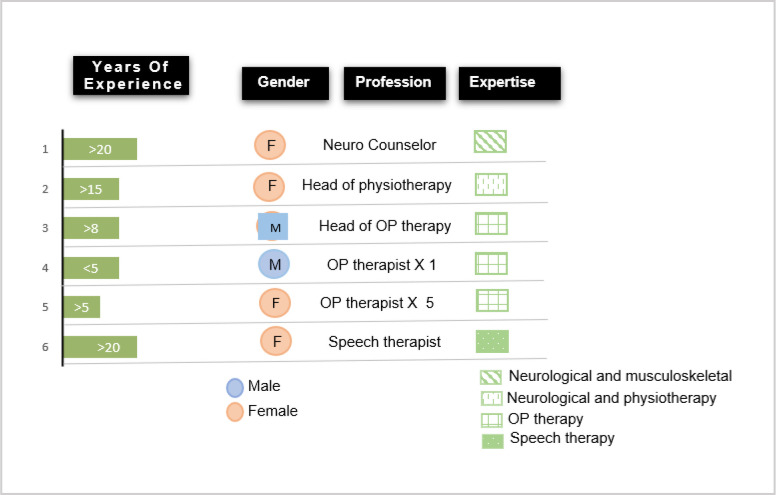
The Project Participants

This study was conducted in a government run rehabilitation center in the city center area of Karachi, which is visited mostly by children living in rural surroundings of the city. Also, there were children visiting from nearby villages and towns and even from cities of other provinces like Balochistan. Our data collection included observation and video recording of many such children from rural areas. Due to lockdown and pandemic protocols, only half of the occupational therapists were available on any given day. Therefore, we selected one group of seven occupational therapists (five females and two males) for our study. Figure 2 provides an overview of the different participants involved from the healthcare organization.

We employed various qualitative data collection methods. These included semi-structured interviews (both in-person and online) and videorecording of the therapy sessions for patients with cerebral palsy visiting the Center. The interviews lasted 30 minutes or more, depending on the availability of the therapists. Data regarding the needs and expectations of the stakeholders were also collected using focus groups, demos, and workshops. We also kept field notes during participatory observation of the therapy process as well as our own development meetings.

The identification details were removed from the field notes, interviews, and focus group data. The collected data were analyzed using thematic analysis. After data familiarization, the corpus was reread to identify preliminary codes. We then grouped codes into relevant sub-themes. The thematic analysis (Braun & Clarke, 2006) helped us understand: (1) the challenges associated with the current in-person communications; (2) how technology can be plugged in to overcome these challenges; (3) the needs and requirements of the end users; and (4) how the acquired capabilities can be targeted toward challenges and communication enhancement initiatives both at individual and organizational level. Table 1 presents an example of an analytical process.

**Table 1:** A Glimpse of Analytical Process

Quotes	Codes	Sub Theme	Themes
Many a times the child needs to be referred to another physician or the child conditions are reversed or deteriorating due to other associated ailments. It is difficult to remember the previous documents or assessments are important, which most of the time are not available. (Therapist F3)	Missing documentation	The need for chronological recording of all relevant documents, reports, tests and planners prepared by the OPD to be followed in different departments.	Towards a documentation and storage tool
The child will remain missing for months and the therapists are not aware for their conditions (Therapist F1)	Multiple chronic ailments	The need for sending alerts to the therapists in a chronological order with system generated timestamps. This should raise numbered notifications for the therapists.	Towards a monitoring tool.
There are many a times that a patient is travelling from interior Sindh or far-flung places and request from us to provide pictures or video therapies and we have none available. In that case we share some photocopies of the exercises but since this is a skill-based activity, it should be taught with some guidelines. (Neuro-counsellor)	Skill based knowledge	Interplay of tacit knowledge of therapists into explicit	Tool that uses different modes of communication (voice and text notes, video)
We often suggest the caregivers to video record the activities but the phone is not accessible to them all the time. (Therapist F1)	Resources scarcity	Create a track of video-based exercises to be assigned by the therapist to the caregiver	Asynchronous solution
My client got divorced from her cousin-husband due to the pressure of giving away the special child to any welfare organization which she did not accept. Important is that we bring change in mindsets and change the culture and associated taboos (Therapist F4).	Stigmatization	Raising hopes through innovation and technology. Every child should have access to treatment.	Increasing reach to patients
The pandemic has highlighted the barriers we face in terms of internet accessibility, WIFI and resources accessibility. We cannot opt for video-based therapies. (Therapist M2)	Wi-Fi availability	The software should allow tracks to be accessed and downloaded anytime. The use of the software requires a low bandwidth making it cost-effective.	Download tracks require low bandwidth.
Since in Pakistan multiple regional languages are spoken, most caregivers can only understand Urdu but speak a different language which we therapists cannot understand (Therapist F1).	Language barriers.	The dashboard of the caregivers is created in Urdu and it also allows the caregivers to give input into the system in Urdu.	Understandable language.
There are workshops being carried each year but mobilizing information and connecting parents are difficult (Therapist M1).	Patients record keeping	Register patients phone numbers, demographic details and conditions. Allowing easy search of patients through their conditions.	Registering and searching patient.
I wish that one day we can have integrated system implemented where different departments and their therapist can share the treatment plan and review the child's progress together (Therapist F5).	Poor healthcare system	Creating a system where data is saved permanently, reducing burden on existing healthcare system and the therapists.	Digitalization of long-term care permanently.
This child is not a regular case. He has a genetic disorder. We cannot assign him the videos from the recorded repository. We need to capture his own video-based tracks (Therapist F1).	Handling special cases	Can help in standardization of therapies for all types of patients, maintain organization memory, and training junior therapists.	Interplay of tacit to explicit knowledge

Based on the above analysis, a prototype app was developed. The app was designed and developed in three phases: constructing HTML pages, developing wireframes of a web app, and implementing a fully functional high fidelity web app prototype. It was important that the end product is usable and accepted by both the groups. PAR has been proven to increase usability of the end products by engaging end-users throughout the study (Bjerkan et al., 2014), therefore, it proved useful for this study.

## Sehat Agahi Web App

The web app supports two interfaces, one for the therapists and one for the caregivers. The therapists' interface provides a separate dashboard for each patient, where the therapist can assign a track, upload documents, view progress, and monitor special events.

A track is a list of exercise videos along with instructions. These tracks are composed of milestone-based exercises prescribed according to the child's development. The web app enables therapists to manage their patients' unique care needs by providing the caregivers with various learning materials in the form of the tracks. The therapists can also see their patients' progress and provide relevant feedback.

**Figure 3:**
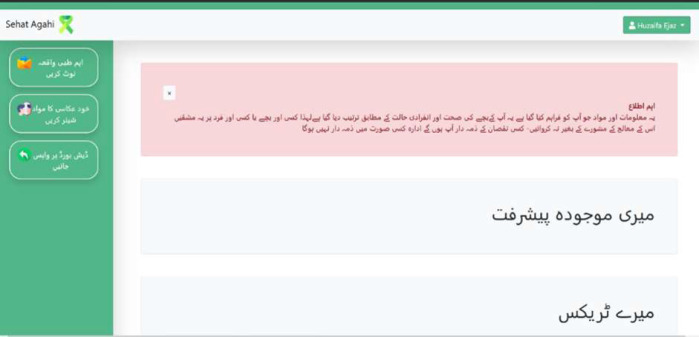
Caregiver's Dashboard with a Ticker Display about Data Privacy

The caregiver's dashboard enables them to see and edit their individual profiles. They can also view or download exercises in their assigned tracks from the therapists. They can record progress of their children, which can then be seen by their therapists. Furthermore, they can notify the therapists of special events, chronic ailments, or other conditions affecting the child. These notifications are accumulated in a system generated log for future data analysis purposes. Since the app contains medical information, to ensure privacy of the content, the lead occupational therapist helped us prepare a ticker to alert the caregivers about data privacy. This text was translated into Urdu and later shared with a legal expert to suggest changes. Following (Figure 3) is the screenshot of the ticker which is displayed as soon as caregivers log in to their dashboard. The following paragraphs describe the web app in more detail.

### Therapist's Home Page

**Figure 4:**
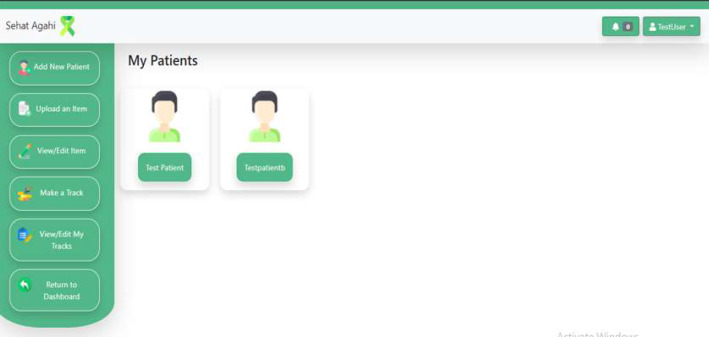
Therapist's Dashboard Home Page

The home page is simple and concise, as shown in Figure 4. The dashboard at the therapist's end is in English, while for caregivers it is in their native language, Urdu. The therapist's name appears in the top right corner. All the services required to support the caregiver appear in the left sidebar of the home page.

### Patient Registration

**Figure 5:**
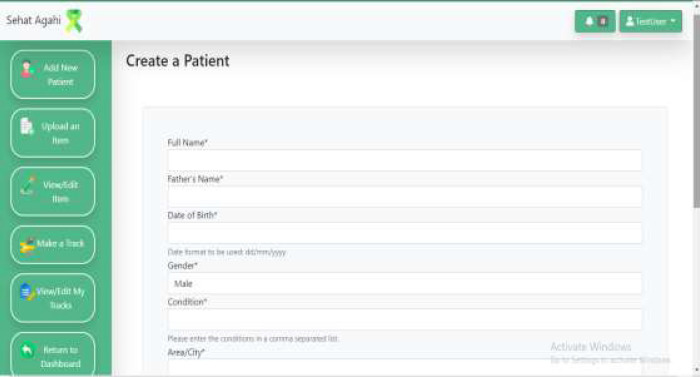
Register Patient

The therapist can register only his/her patients. The registration page contains data related to the child's age, condition, area of residence, and phone number of the caregiver as shown in Figure 5. The registration process is usually performed during the therapy interaction; therefore, it is designed to be effortless and take only a few minutes to complete. More information can be added anytime, as these children usually suffer from many associated ailments. Also, a child's condition may relapse to a previous milestone due to various reasons. Once registered, the name and icon of the patient appears on the homepage of the therapist's dashboard. Upon clicking the icon, the patient's home page (discussed below) opens for the therapist to complete related tasks.

### Uploading an Item

Therapists can upload and manage items like documents, images and videos related to the attainment of different milestones and positive behaviors of the child. This repository is updated on a continuous basis. These activities and therapies are stored in a standardized manner by combining the name of the exercise with the name of the related body part and the condition of cerebral palsy, so that different therapists can search for and use the therapies for their own patients as suitable.

### Preparing Tracks

Therapists can combine different video exercises, activities, documents, or images to create a track of items to be shared with patients as required according to their needs and conditions. A track can be used by multiple therapists from the database and can be assigned to multiple children with similar conditions.

### Assigning a Track to a Patient

Upon clicking the patient icon, the therapist is routed to the patient's individual page. Here, the therapist can assign multiple tracks relevant to a child's needs at the time. These can be accompanied by instructions like exercise duration, alternate resources that can be used in exercises, indications of similar activities in daily life, or special instructions and precautions. These instructions can be both voice and text based, depending on the caregivers' learning needs.

### Monitoring Progress

The therapist can view exercise dosage on the main page of a child on a weekly basis. The number of activities assigned will be tabulated week-wise to show how many times the caregiver administered the exercises to the child. Due to low education and technological literacy, the weekly tracks seen and practiced are reported automatically to the therapists to supervise and manage progress.

### Uploading Reports and Labs

**Figure 6:**
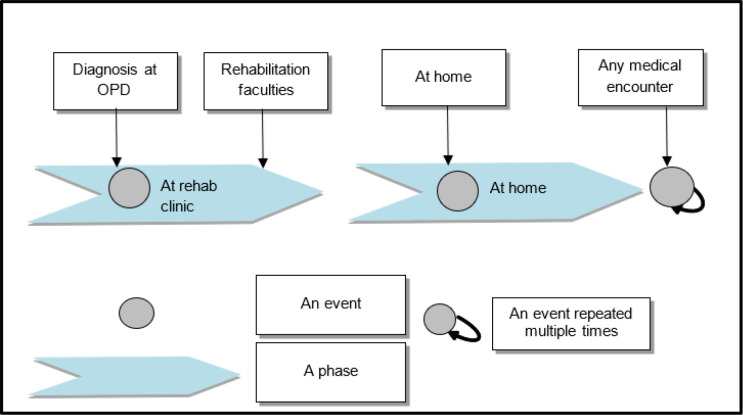
Important Events/reports Generation

Interviews with therapists and caregivers revealed that many times important documents are missing when required. This component of record keeping is also the responsibility of the therapist. A significant amount of knowledge is generated in the form of therapy documents at various phases of the long-term rehabilitation journey of the child daily, as depicted in Figure 6. The caregivers record significant events, while the rest of pertinent data are recorded by the therapists. The diagnosis report, recommendation, labs/tests, associated medical documents, and records of treatments and medicines for the different associated chronic ailments are all uploaded to the patient's record. The therapists can share this with the other departments when requested.

### View Special Events

The therapists can use a special feature through which they can keep up with the child's condition, especially chronic medical conditions. This feature works by accumulating notifications from the caregivers and updating the log. For example, if a child falls sick with fever, the caregiver can enter the child's symptoms. The system then automatically sends a notification and updates the log at the therapist's home page.

## Discussion

Capacity building is a dynamic and complex process involving rethinking service design using knowledge from different domains. Our study explored how technology can help in improving communication to develop parental competency. In the following paragraphs, we will discuss how the technology solution features impacted the design of healthcare services.

### Capacity Building Features

This section describes various features of the app and discusses how they help communication at different levels of the healthcare organization, including individual, team, and systems levels.

### Sehat Agahi Supports Caregivers' Learning

This app allows for continued communication between caregivers and therapists. The two-way communication allows participation in diagnosis, training, monitoring, and problem-solving. For example, the therapists can share a digital long-term plan for the child with the caregivers, who can implement it, and reflect on the shared care plans. As one of the caregivers, when returning to the therapy centre after a long break reported, “My daughter was born five months back; it was really difficult to bring Amin (not her real name) to the center during that phase. The technological solution will help support Amin at home, minimizing the needs of frequent travelling, which seem difficult with a young born.”

This supports skill-building in caregivers using a training and supervision model. The app taps into the expertise of the therapists and applies the acquired technological and strategic capabilities to improve knowledge and skills of caregivers. This improved communication can help in internal capacity building at individual and team levels, and in the overall capacity of the healthcare organizations.

### Sehat Agahi Helps Service Redesign Based on Caregivers' Needs

The system supports caregivers in learning the techniques for assisting in therapy. They can supplement their direct learning acquired through in-person meetings with indirect learning acquired through the app. As one of the mothers reported, “As Arshad (not his real name) enters the center he starts to cry, his challenging behaviors disrupt my learning. It is always easy to go back at night in my free time and catch up the missing lessons.”

The app supports caregivers by mitigating the barriers associated with knowledge transfer and learning. These barriers include socioeconomic, geographical, and cultural barriers such as travelling, living in rural settings, (Hamdani et al., 2014) and dealing with challenging behaviors of children (Cason, 2011; Langkamp et al., 2014). This type of learning is also termed as single loop learning in the literature (Lim et al., 2015). Since the app provides on-demand facilitation, overtime the caregivers can begin to generalize the skills and expertise gained using instructions to work in dynamic and natural environments with the child. This would enable them to adapt to the changing needs of their child. This increased capacity of caregivers integrated within the system increases the overall capacity.

### Sehat Agahi Aids in Delivering on Quality and Cost

**Figure 7:**
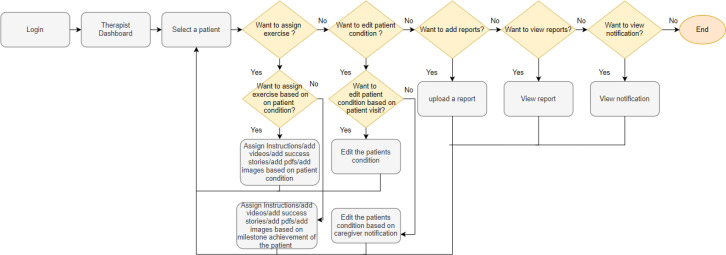
Service Redesign

Integration of a digital app such as Sehat Agahi in a healthcare ecosystem also helps in reducing costs, increasing reach and service delivery as well as improving the quality of service provided. The app allows the therapists (see Figure 7) to not only entertain those patients who visit the rehabilitation center regularly, but also patients who can only visit once a year or so (Hamdani et al., 2014). As one of the families visiting from Quetta city reported, “This is first time in three years' time that we are visiting this rehabilitation center. We were directed by a Karachi-based pediatric doctor to this center. In Quetta, we don't have such a facility. But it will be difficult to come back again frequently.” Similarly, this platform allows therapists to work from home, thus increasing their productivity. Many female therapists in Pakistan leave the practice due to cultural, social, or individual reasons (Masood, 2019). This app helps such therapists by overcoming or sidestepping such barriers. In addition, since the app maintains a history of the child's plans and progress, it minimizes the disruption caused by a female therapist going on a long maternity leave.

## Capacity Building in Organizational Context

In this section we discuss the ways in which this app improves communication and thus enhances capacity building within healthcare organizations.

### Knowledge Exchange and Interplay of Tacit and Explicit Knowledge

Sehat Agahi enables process and service redesign using technology-based skill development in the therapists as a springboard. For example, the item repository, which contains content uploaded by all the therapists, allows for a quick search for the relevant content to be shared with the patients. Since it is a common repository, it encourages learning in groups. The therapists can learn how other therapists treat their patients, and what therapies and activities work better in different situations. This leads to discussion and knowledge exchange on the team and helps the organizational memory to grow. If knowledge remains with individual therapists and is not shared, organizational learning is hampered.

The items and tracks assist in explicit codification of procedures, and standardization of therapeutic processes, leading to formalization at the system level. This also helps in training young therapists (i.e., helping them study individual cases by giving them access to case history, the results of previous tests, prescriptions and related documents, and outcomes). The video-based tracks can help young therapists understand the activities and exercises related to the attainment of physical milestones and positive changes in behavior. The app also provides access to informational readings, and audio and written notes to establish context for the therapy. Such knowledge exchange and codification can also easily lead to improvement and redesign of curricula based on accumulated experience and building on organizational memory.

### Repeated Practice Leads to Evolution of Capabilities

Sehat Agahi helps therapists integrate technology in their daily routines and practices. For example, during the in-person therapy sessions, the therapists can use technology for transforming the inputs (e.g., items, tracks) to outputs (e.g., sharing a care plan with a caregiver or storing important reports). By doing so each day as a routine, they develop their digital skills. Also, the record keeping on a day-to-day basis of child-patient reports, history, and significant events, enables the therapist to accomplish educating and supervising, which leads to formalization of protocols and standards. This repeated practice of ordinary capabilities could lead to the evolution of capabilities and sustainable change.

Sehat Agahi helps in evolution of low-order capabilities into high-order capabilities. Low-order capabilities include simple functions such as patient record keeping (e.g., profiles and reports), maintaining libraries of items and tracks (e.g., videos, audios, images, and documents), standardization of the digital care plans, training and supervision of caregivers and monitoring alerts and caregivers' shared reflection of child's everyday conditions. While these are everyday routine activities, these capabilities can be transformed or integrated into higher order capabilities (Kislov et al., 2014). For example, the integration of these capabilities can help facilitate and implement public awareness campaigns or outreach activities.

Information from Sehat Agahi can help in connecting parents of children having similar conditions, to run campaigns. For example, we can raise dental hygiene awareness by conducting workshops for caregivers of children with quadriplegia. As another example, data analytic techniques can be run on the accumulated database by experts to gauge the effectiveness of particular therapies or exercises on children under different conditions.

### Combining Acquisitive and Experiential Learning

Apart from practice-based experiential learning (where the therapists learn techniques from each other, and accumulated experience), the app supports continuous acquisitive learning from external industry, IT experts, and the caregivers. The caregivers' reflection on attaining milestones can lead to understanding how different therapies impact the child's progress. This element of communication for building internal knowledge bases and improvement of skills leads to an increase in overall capacity of the healthcare system.

### Capacity Building at Individual, Team, and System Levels

The intervention helps not only at individual level; this solution translates into service advancement strategies and disseminating it into group and organizational levels. The app helps to support communication at meso levels and diffuse team conflicts (Lim et al., 2015). By using shared resources in the library for initiating efforts for a child's integrated plan (intra and inter department communication), the therapists not only learn effective therapies, but they also learn skills from expert therapists. The app can connect team members allowing them to access digital medical resources, discuss diagnosis, co-treatments, results, and recommendations. This enables intra-department diagnosis and discussion, leading towards a coordinated health plan for the patients. On a macro level, promoting telehealth and knowledge transfer can lead to overall advancements in the healthcare system.

## Limitations

Sehat Agahi is designed to support appropriate telerehabilitation services that include an asynchronous mode of communication which does not require high speed data as required by video streaming based telerehabilitation services. However, the caregiver requires a smart phone and cellular data packages for this service. We also found that there is usually one smart phone available per family, therefore flexibility of time of access to exercises and download option was provided for ease. The socioeconomic conditions of the caregivers demanded a cheaper solution, as most caregivers do not have large screen laptop or tablets; this might compromise their learning. Currently, we are storing best practices of the therapists in the form of items which contain no identifying metadata of the child-patient, so privacy is preserved to a large extent. However, in some of the videos stored in the database, the face of the child-patient may be partially or fully visible. This is a current limitation. However, we have plans to automatically blur the faces in the future versions of the app.

## Conclusion

Sehat Agahi is a smart phone-based communication solution to diagnose, teach, supervise, and track the care of developmentally disabled children. This solution facilitates asynchronous communication, thereby mitigating the provider shortage problems in under-served areas of Pakistan to some extent. Sehat Agahi is a technology-based, therapy provision intervention, designed to mitigate various demographic barriers to communication faced by both the caregivers and therapists.

The app also serves as a learning mechanism for the caregivers. This enhances the caregivers' capacity to care for their child by mitigating the various communication barriers explored during the study. With continued success of caregiver participation and integration into the healthcare system, it is hoped that the overall capacity will generally increase. The use of this app will eventually enable further digitization of healthcare and enhance its capabilities and equity.
